# Rare *c-KIT* c.1926delA and c.1936T>G Mutations in Exon 13 Define Imatinib Resistance in Gastrointestinal Stromal Tumors and Melanoma Patients: Case Reports and Cell Experiments

**DOI:** 10.3389/fmolb.2022.730213

**Published:** 2022-06-02

**Authors:** Chi Yan, Chengzhi Zhao, Ke Yang, Hongyan Zhou, Limin Jing, Weixing Zhao, Wenguang Dou, Qingxin Xia, Jie Ma, Bing Wei, Yongjun Guo

**Affiliations:** ^1^ Department of Molecular Pathology, The Affiliated Cancer Hospital of Zhengzhou University and Henan Cancer Hospital, Zhengzhou, China; ^2^ Henan Key Laboratory of Molecular Pathology, Zhengzhou, China; ^3^ Department of Pathology, Xinxiang First People’s Hospital, Xinxiang, China; ^4^ Computed Tomography Room, Xinxiang First People’s Hospital, Xinxiang, China; ^5^ Department of Pathology, The First Affiliated Hospital of Xinxiang Medical University, Xinxiang, China; ^6^ Department of Radiology, The First Affiliated Hospital of Xinxiang Medical University, Xinxiang, China; ^7^ Department of Pathology, The Affiliated Cancer Hospital of Zhengzhou University and Henan Cancer Hospital, Zhengzhou, China

**Keywords:** GIST, c-KIT, melanoma, rare mutations, imatinib resistance

## Abstract

**Background:** Target therapies play more and more important roles in gastrointestinal stromal tumors (GISTs) and melanoma with the advancement of clinical drugs that overcome the resistance caused by gene mutations. *c-KIT* gene mutations account for a large portion of GIST patients, which are known to be sensitive or resistant to tyrosine kinase inhibitors. However, the role rare mutations play in drug efficacy and progression-free duration remains elusive.

**Methods:** Two rare mutations were identified using Sanger sequencing from the GIST and melanoma cases. Cell experiments were further carried out to demonstrate their role in the imatinib resistance.

**Results:** *c-KIT* c.1926delA p.K642S*FS mutation in primary and recurrent GIST patients and *c-KIT* c.1936T>G p.Y646D point mutation in melanoma patients in exon 13 were first demonstrated to be novel targets resistant to imatinib agent.

**Conclusion:** *c-KIT* mutations c.1926delA and c.1936T>G in exon 13 are clinically significant targets that exhibit resistance to imatinib. This study provides guidance to GIST and melanoma treatments.

## Introduction

Gastrointestinal stromal tumors (GISTs) are the most common mesenchymal malignancy of the gastrointestinal tract, with an incidence of 7,000–8,000 cases per year in China (Yuval and Elazary, 2018). Over 90% of GISTs stained positive for c-KIT by immunohistochemistry, and around 70–85% of tumors had a functional *c-KIT* mutation by sequencing technology (Lee et al., 2013; Shi and Liang, 2014; Li and Raut, 2019). Using multi-gene panels by NGS, *APC*, *CDH1*, and *TP53* mutations were found in multiple hereditary cancers such as gastric cancer and colorectal cancer (Slavin et al., 2015); however, they were not the priority in GISTs. Typically, *c-KIT* and *PDGFRA* gene mutation tests were recommended to assist molecular diagnosis and target therapy by the guidelines, and other mutations would be considered, given the absence of known positive variations. Over 60% of *c-KIT* alterations in GISTs are located in exon 11, followed by exon 9, which encodes the juxtamembrane domain responsible for inhibiting receptor dimerization and activation in the absence of the SCF ligand (Tan et al., 2018). Less common mutations have also been reported in exons 13, 17, or other parts of *c-KIT*, although they are usually regarded as secondary mutations resulting from tyrosine kinase inhibitor actions (Kobayashi et al., 2015; Nakano et al., 2017).

*c-KIT* mutations were found not only in GIST but also in melanoma patients (Mazurenko et al., 2017). *c-KIT*-mutated melanoma represented various frequencies in different populations and histological types, with the highest rate in Asians (Moltara et al., 2018; Gutiérrez- Castañeda et al., 2020). *c-KIT* mutations were present in 36% of acral lentiginous melanomas in cutaneous melanoma (Kong et al., 2011; Gutiérrez- Castañeda et al., 2020). Approximately 70% of mutations were identified to reside in exons 11 and 13 of the *c-KIT* gene, of which the commonest one was L576P for the former and K642E for the latter (Reddy et al., 2017; Davis et al., 2018). These mutations led to the activation of MAPK and other cell signaling pathways causing tumor cell proliferation and cancer progression (Calipel et al., 2014). Thus, activating *c-KIT* mutations act as the target of clinical therapies in melanoma (Jiang et al., 2008). However, it remains to be demonstrated whether the rare c-KIT alterations are effective to tyrosine kinase inhibitors in malignant melanoma.

Considering the crucial roles of *c-KIT* in GIST and melanoma tumorigenesis, targeted inhibitors were thus used as the treatments for *c-KIT*-positive patients. The most widely investigated *c-KIT* inhibitor was imatinib, which served as the first-line drug in GIST (Blay et al., 2015), whereas as an alternative treatment beyond immunotherapy in melanoma (Carvajal et al., 2011; Guo et al., 2011; Delyon et al., 2020). For patients with resistant *c-KIT* mutation, a second-generation tyrosine kinase inhibitor such as nilotinib was proven more efficient than imatinib in GISTs and metastatic melanoma (Tran and Tawbi, 2012). Although somatic mutations in *c-KIT* were present over the entire gene, it appeared to be different for the efficacy of c-KIT inhibitors in patients owning mutations residing in exons 11 and 13. Improvement of the efficacy of c-KIT inhibition will require a better understanding of the mechanisms underlying response and resistance to treatments (Jiang et al., 2008). The roles of the novel mutations in exon 13 of the *c-KIT* gene in the tolerance to imatinib in GISTs or melanoma remain uncertain.

In this study, it is found that *c-KIT* c.1926delA p.K642S*FS mutation in primary and metastatic tumors of the GIST patients and c.1936T>G p.Y646D point mutation in melanoma cases were rare targets resistant to imatinib mesylate, which was identified using Sanger sequencing and cell culture experiments. This study was capable of providing valuable treatment or diagnosis information for GIST and melanoma patients harboring either mutation.

## Materials and Methods

### Treatment of the Gastrointestinal Stromal Tumors Patient

Patient 1, a woman at the age of 49, was first diagnosed with gastric GISTs at Xinxiang First People’s Hospital in August 2017 using a biopsy specimen of the primary tumor from the stomach. After imatinib treatment for approximately four and a half months, there was a symptom of disease progression, and thereafter, the primary tumor was resected by surgery in the First Affiliated Hospital of Xinxiang Medical University at the end of December 2017. The surgical specimen of the primary tumor was pathologically diagnosed as gastric wall plexiform fibromyxoma. Postsurgical drugs were not taken until the new tumor was present at the right bottom of the stomach during the routine follow-up examination around seven and a half months later. Subsequently, the patient was orally treated by Gleevec for 1 year until the second resection of the metastatic tumor in the Affiliated Cancer Hospital of Zhengzhou University. All the pathological sections obtained by hematoxylin–eosin staining and immunohistochemistry were reviewed by two pathologists.

### Treatment of the Melanoma Patient

Patient 2, a man aged 69 years, suffered from a metastatically malignant melanoma at the right thigh that originated from the primary tumor at the bottom of the right foot and had a novel mutation in exon 13 of the *c-KIT* gene with the polymorphic alteration of c.1936T>G and amino acid change p.Y646D as well as the wild-type PDGFRA. None of the c-KIT-targeted treatments were performed on this patient, except that he was prescribed 10 cycles of immunotherapy for anti-PD-1 antibody.

The retrospective study was approved by the Ethics Committee of the Affiliated Cancer Hospital of Zhengzhou University according to the 1964 Helsinki Declaration and its later amendments or comparable ethical standards. Written informed consent was obtained by both patients.

### c-KIT Mutation Analysis

The formalin-fixed and paraffin-embedded specimens from the biopsy and the surgeries of both patients were sectioned, and genomic DNA was isolated by using a kit (Qiagen, Cat. No. 56404). As the leukocytes from both patients were not preserved in the regular physical examination, the FFPE tissues after surgery were taken from the normal part of the patients. The normal marginal counterparts matched with the tumor FFPE tissues that were resected from the two patients were sectioned to isolate the genomic DNA as well. *c-KIT* mutations in exons 9, 11, 13, and 17 and *PDGFRA* mutations in exons 12 and 18 were analyzed by using the commercial kits (Yuanqi Bio, Shanghai) including primer pairs used to amplify the entire sequence for each exon. Primer sets (forward: 5′-CAC​CCT​GTT​CAC​TCC​TTT​G-3′, reverse: 5′-GGT​ATG​TCC​TGG​GCT​GTT​C-3′) were employed to amplify the regions spanning exons 11 and 13 to determine the relative location of different variations on the same chromosome. The PCR amplicons were sequenced using Sanger sequencing.

### c-KIT Tyrosine Phosphorylation Analysis

The wild-type and mutated c-KIT sequences with a notation of WT, M1, M2, M3, and M4 (Table 1) were cloned into the expression vectors with the GFP tag, as reported previously (Isotani et al., 2010). Specifically, the total length of the c-KIT coding sequence was amplified and ligated to the mammalian expression vector pcDNA3.1 (+) tagged with the GFP coding sequence at its 5′ terminus. The site-directed mutants of c.1668_1679delGTGGAAGGTTGT and c.1926delA as well as c.1936T>G were introduced to the wild-type expression plasmid.

**TABLE 1:** Genomic changes of the *c-KIT* gene for plasmid construction.

c-KIT coding sequence	Genomic change
WT	none
M1	c.1668_1679delGTGGAAGGTTGT
M2	c.1668_1679delGTGGAAGGTTGT/c.1926delA
M3	c.1936T>G
M4	c.1926delA

c-KIT tyrosine phosphorylation was detected, as published previously (Nakano et al., 2017). In brief, SK-MEL-28 cells were plated at a density of 5 × 10^5^ cells on the 6-well plate. After cell adherence, c-KIT-GFP expression vectors producing wild-type c-KIT or the c-KIT with mutated c.1668_1679delGTGGAAGGTTGT or both, and c.1936T>G were transiently transfected into these cells using Lipofectamine 2000 Reagent (Invitrogen, Cat. No.11668-019). After growing for another 30 h, these cells were treated with or without 100 ng/ml human stem cell factor (SCF, PeproTech, Cat. No. 300-07) for 20 min. In order to analyze the tyrosine kinase inhibition, the cells were treated with imatinib mesylate at concentrations of 0.01, 0.1, 1, and 10 μM, individually. The harvested cells were subject to Western blotting using monoclonal mouse anti-human c-KIT antibody (Santa Cruz, Cat. No. sc-13508) and rabbit anti-human phospho-c-KIT antibody (Thermo Fisher Scientific, Cat. No. 44-490G), as well as monoclonal mouse anti-human phosphotyrosine antibody (Millipore, Cat. No. 05-321X).

### Cell Proliferation and Apoptosis Assay

MTT assay was employed to evaluate cell proliferation. Briefly, after being transfected by plasmids WT, M3, or M4, SK-MEL-28 cells were incubated for 30 h. Then, the cells were digested and plated on the 96-well plate at a density of 5 × 10^3^ per well, followed by the imatinib treatment for 24 h or 48 h at concentrations of 0, 20, 40, 80, and 100 μM, respectively. At the individual time point, 10 µl of the stock MTT solution (5 mg/ml, MCE, Cat. No. HY-15924) was added to each well, and the cells were incubated for 4 h at 37°C. Then the supernatant was discarded, and 150 µl of DMSO was added to dissolve the formazan crystal produced by the cells. Finally, the absorbance was determined at a 568-nm wavelength on a microplate reader.

The proliferation rate was equal to the percentage of the mean OD from each treated group divided by the mean OD from MOCK. A remarkable difference between the two groups was analyzed by Student’s t-test. A *p*-value < 0.05 was considered statistically significant. Each data group was representative of three replicates unless otherwise indicated.

Cell apoptosis assay was performed using the Annexin V-APC/7-AAD Apoptosis Detection Kit (KeyGEN BioTECH, Cat. No. KGA 1026). Specifically, the cells were transfected by plasmids WT, M1, M3, or M4, followed by incubation at 37°C for 30 h. Then 100 μM imatinib was added to the cells, which were cultured for another 72 h. Subsequently, the cells were collected and further resuspended by 500 μl binding buffer. Next, 5 μl Annexin V-APC and 5 μl 7-AAD were sequentially added to the cell suspension. Finally, the samples were examined by flow cytometry (BECKMAN) after incubation for 5–15 min at room temperature under dark conditions.

## Results

### Medical Imaging and Immunohistochemistry of the Gastrointestinal Stromal Tumor Patient

**FIGURE 1:**
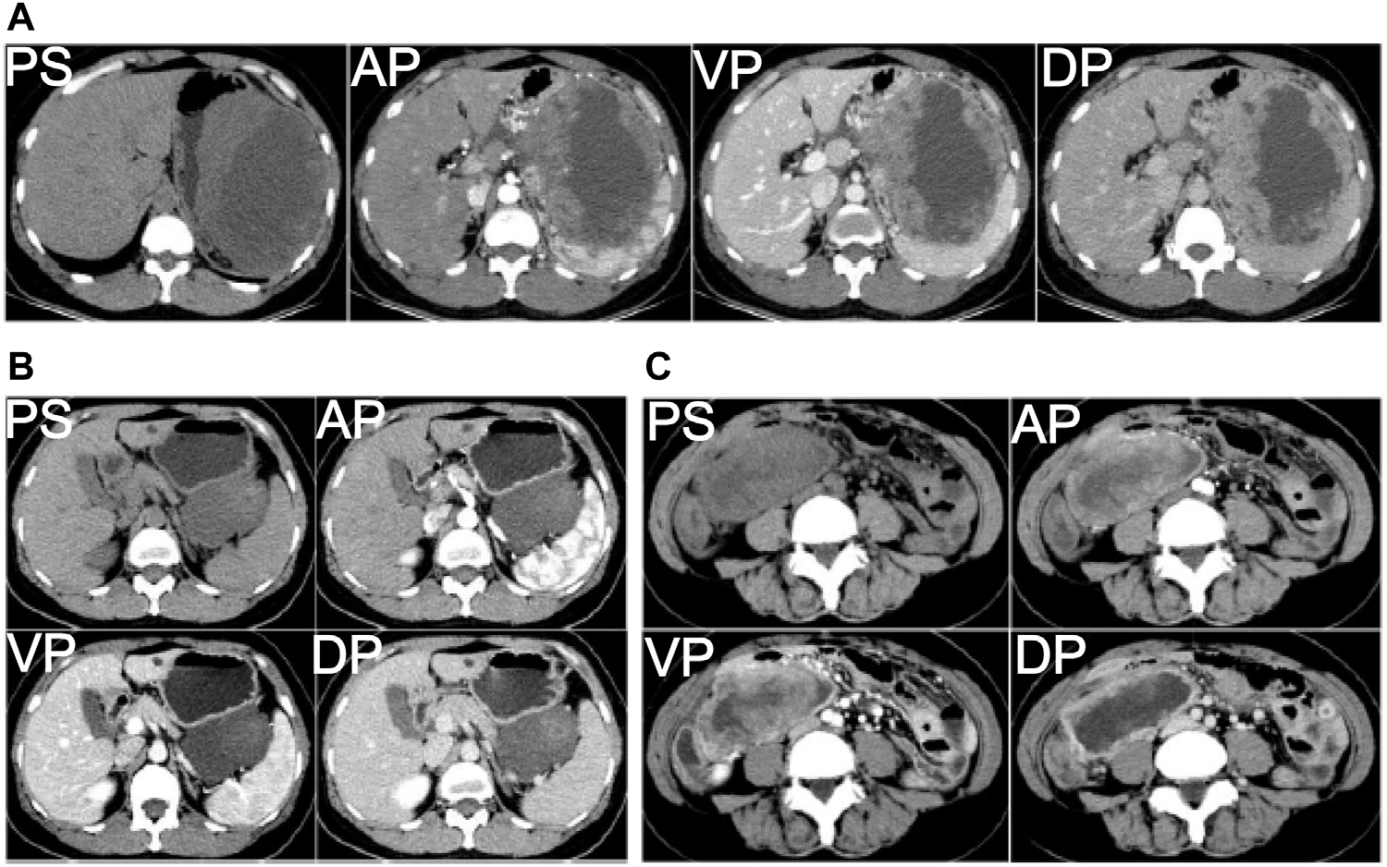
CT imaging of the female patient at different phases of the tumor progression. **(A)** CT imaging of the female patient at the initial diagnosis in the Xinxiang First People’s Hospital (photo taken on 2017-08-06). **(B)** Primary tumor shrinking after imatinib treatment for around four and a half months (photo taken on 2017-12-27). **(C)** Recurrent tumor was observed seven and a half months after primary tumor resection (photo taken on 2018-08-13). PS: plain scanning, AP: arterial phase, VP: venous phase, DP: delay phase.

To make it clear what caused the stomachache of the female patient, enhanced computed tomography (CT) was carried out, and a biopsy specimen of the primary tumor was obtained from her stomach in the Xinxiang First People’s Hospital. The imaging results showed that the bottom of the stomach was occupied by lumpy soft tissue density shadow, with the likelihood of the GISTs (Figure 1A). To further prove this, immunohistochemistry was performed using the biopsy specimen. The data indicated that CD117 and CD34, as well as DOG-1 and Ki-67, were positively stained with negative expressions of SMA and S-100 proteins (Table 2); thus, the patient was diagnosed with gastric gastrointestinal stromal tumor.

**TABLE 2:** Clinical and pathological characteristics in the GIST patient.

Pathological feature	Primary tumor		Recurrent tumor
	Biopsy specimen	Surgical specimen	
Tumor size	NA	8 × 5 × 5 cm	7 × 6 × 6 cm
Histology			
Cell morphology	Spindle	Plexiform	Spindle
Mitosis/50 HPF	NA	NA	>50
Immunohistochemistry			
CD117	+	−	+
CD34	+	−	+
DOG1	+	−	+
SMA	−	+	+
Ki-67	+ (10%)	+ (1%)	+ (60%)
S-100	−	−	+ (sporadic)
*c-KIT* mutation			
Exon 11	+	+	+
Exon 13	+	+	+

NA indicates “not available.”

After imatinib treatment for four and a half months based on the mutated *c-KIT* gene in the biopsy specimen, the primary tumor shrank (Figure 1B), followed by surgical resection of the tumor at a size of 8 × 5 × 5 cm (Table 2). Immunohistochemistry staining showed that CD117, CD34, DOG-1, and Ki-67 were expressed at a negligible level. As *c-KIT* and *PDGFRA* gene sequencing was not performed then, this specimen was thus diagnosed as gastric wall plexiform fibromyxoma based on its morphology. However, the patient relapsed seven and a half months later, and the tumor metastasized to other parts of the stomach (Figure 1C). The metastatic tumor was excised in the Affiliated Cancer Hospital of Zhengzhou University at the size of 7 × 6 × 6 cm and was pathologically diagnosed with GISTs in combination with the mutation status of the *c-KIT* gene (Table 2).

### Mutational Analysis on *c-KIT* and *PDGFRA* Genes for the Gastrointestinal Stromal Tumor patient

**FIGURE 2:**
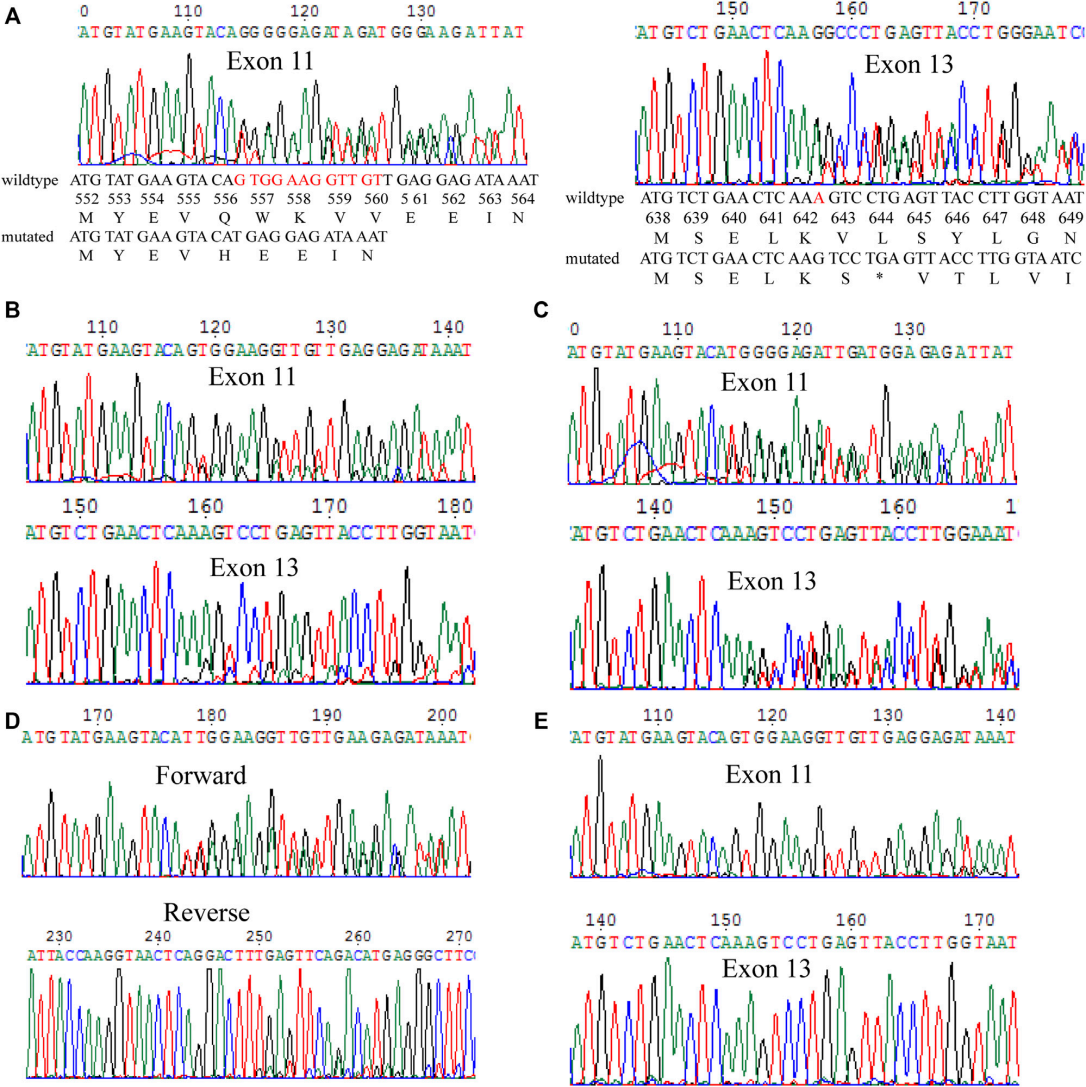
Mutation analysis of the *c-KIT* gene in different specimens. **(A)** Biopsy specimen of the primary tumor. *c-KIT* c.1668_1679del GTGGAAGGTTGT mutation in exon 11 and c.1926delA mutation in exon 13 were detected. The mutated nucleotides are shown in red colors. **(B)** Primary tumor after surgical resection. The same mutations were found in this specimen through biopsy. **(C)** Metastatic tumor after surgical resection. Similarly, the two mutations were detected in this metastatic tumor. **(D)** Detection of the two mutations in surgically primary tumor specimen in a transacting manner. **(E)** Normal marginal tissue matched with the recurrent tumors. The two mutations showed a wild type in the normal tissue.

To determine whether the patient was sensitive to imatinib treatment, the mutational status of *c-KIT* and *PDGFRA* genes was analyzed on the biopsy specimen of the primary tumor by direct sequencing. The data indicated that partial deletion mutation of *c-KIT* c.1668_1679delGTGGAAGGTTGT causing the amino acid loss of p.557_560delWKVV and gain of p.Q556H mutation was present in exon 11, as well as a second novel single-nucleotide deletion mutation of c.1926delA in exon 13, resulting in the production of the stop codon (p.K642S*FS) (Figure 2A). The *PDGFRA* gene showed a wild-type genotype (not shown). All the aforementioned data suggested that the patient could benefit from the imatinib treatment, despite the unclear significance of this frameshift alteration in c-KIT exon 13.

Additionally, the surgically primary tumor specimen and the metastatic tumor specimen were sequenced, and it was discovered that mutations of *c-KIT* c.1668_1679delGTGGAAGGTTGT in exon 11 and c.1926delA in exon 13 were present in both the surgically primary and metastatic tumors (Figures 2B,C, respectively). To further clarify if both deletion mutations were arranged in a *cis* or *trans* manner, PCR was used to simultaneously amplify the fragment in the forward and reverse directions. Results showed that both deletion mutations act in a *trans* manner (Figure 2D). Also, the sequencing map showed that no mutations were detected in genomic DNA from the normal marginal tissue for both *c-KIT* and *PDGFRA* genes, which indicated the two mutations in *c-KIT* exon 11 and exon 13 found in this GIST patient were somatic (Figure 2E).

### Presence of a Novel Mutation in c-KIT Exon 13 for the Melanoma Patient

**FIGURE 3:**
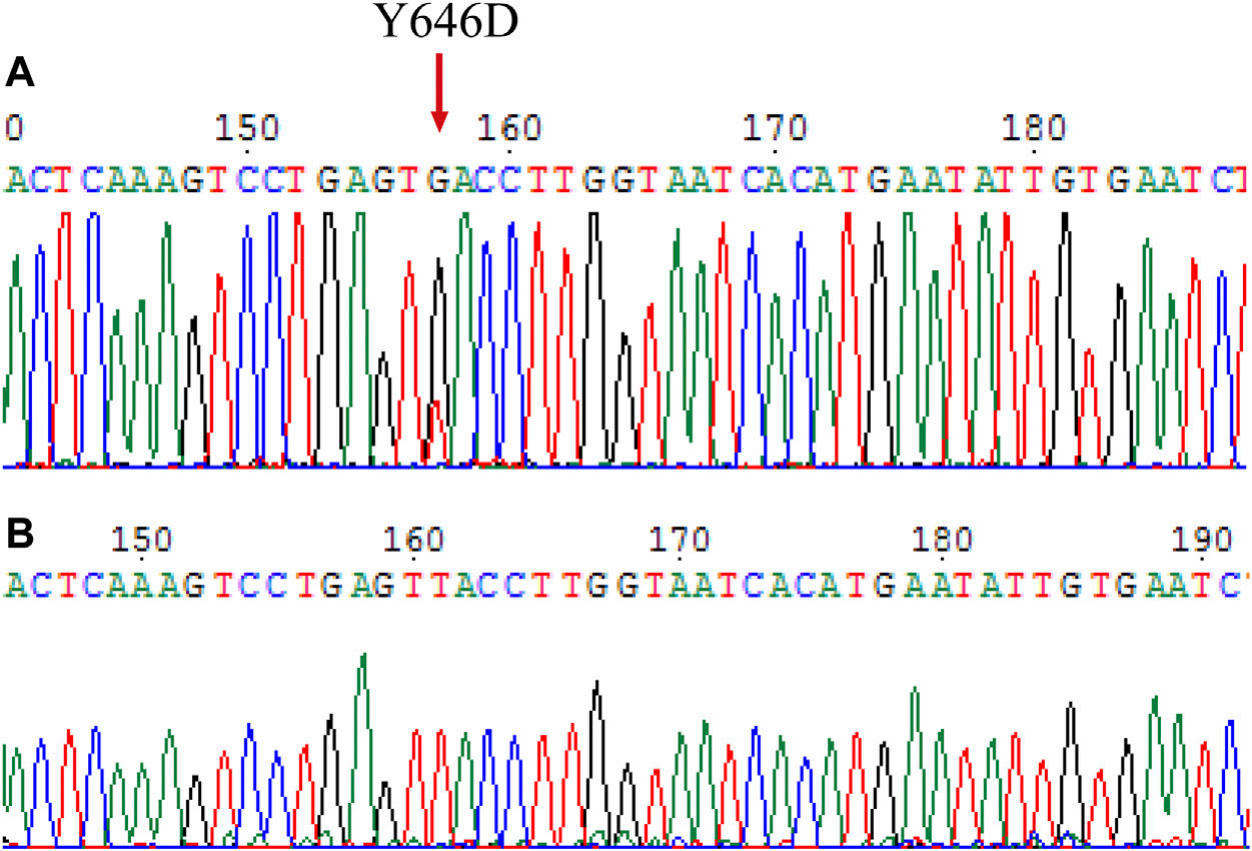
Mutation analysis on the *c-KIT* gene for surgical melanoma specimen. **(A)** Tumor FFPE tissue. **(B)** Normal marginal tissue.

One year after the initial surgical resection of the melanoma from the right foot, patient 2 was found to show metastatic melanoma in his right thigh, and the genetic mutations of *c-KIT* and *PDGFRA* showed that a novel mutation c.1936T>G in *c-KIT* exon 13, causing the production of missense amino acid mutation p.Y646D (Figure 3A). In addition, *c-KIT* exhibited a wild type in normal marginal tissue (Figure 3B), suggesting this *de novo* mutation was a somatic variant. The *PDGFRA* gene had a wild-type genotype (not shown). It was not clear whether this mutation was sensitive to tyrosine kinase inhibitors.

### Two Rare Mutations in c-KIT Exon 13 Were Imatinib-Resistant

**FIGURE 4:**
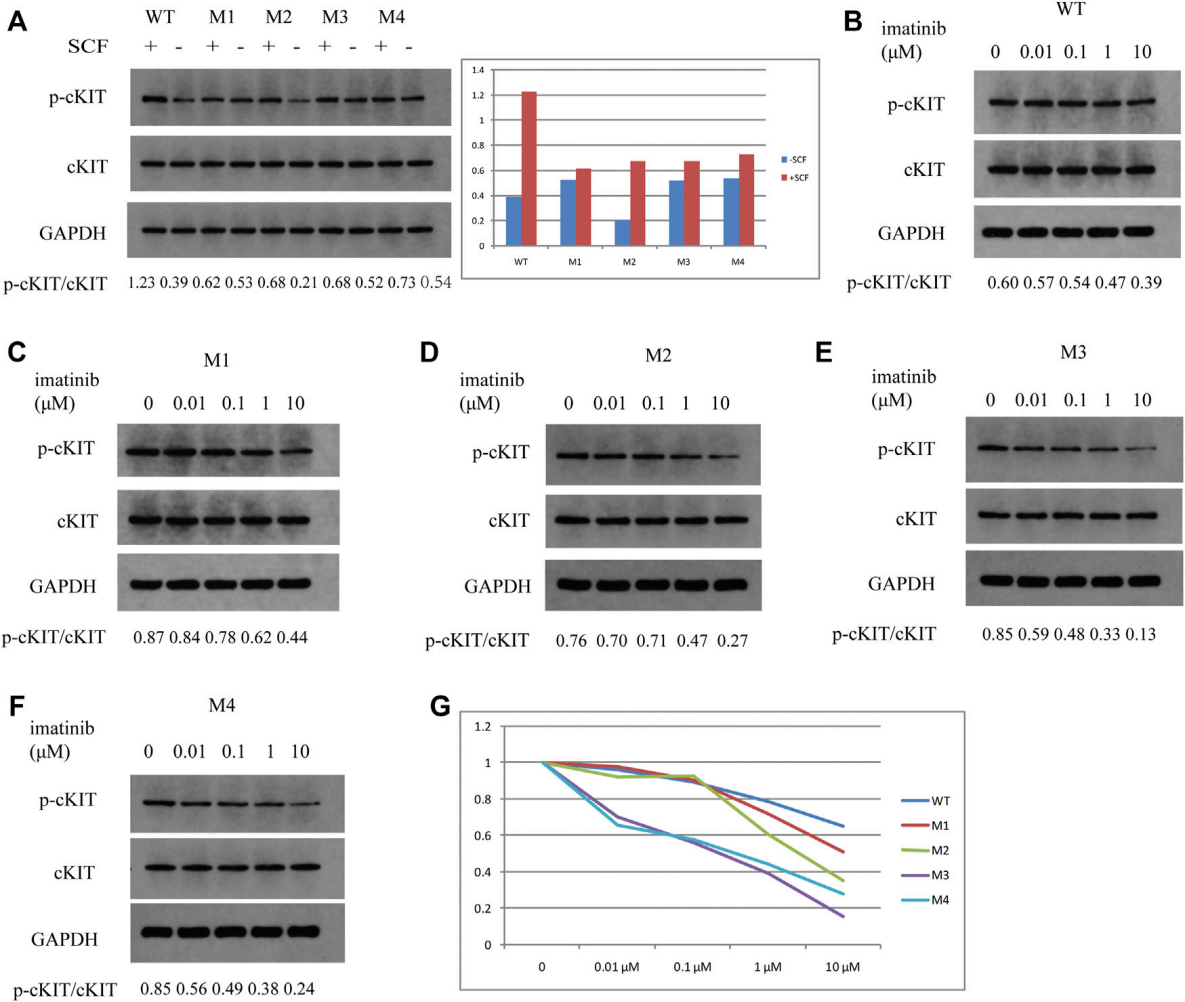
Western blotting detection in SK-MEL-28 cells treated by SCF or imatinib at different concentrations for each plasmid. The ratios of p-cKIT to cKIT accurate to 0.01 are shown at the bottom of each panel. **(A)** c-KIT and phospho-c-KIT levels after SCF treatment for wild type, M1, M2, M3, and M4. The values of p-cKIT to cKIT are shown in the bar chart for these plasmids treated (+SCF) or not treated by SCF (-SCF). **(B)** c-KIT and phospho-c-KIT levels in wild-type-transfected cells after imatinib treatment at concentrations of 0, 0.01, 0.1, 1, and 10 μM. **(C)** c-KIT and phospho-c-KIT levels in M1-transfected cells after imatinib treatment at concentrations of 0, 0.01, 0.1, 1, and 10 μM. **(D)** c-KIT and phospho-c-KIT levels in M2-transfected cells after imatinib treatment at concentrations of 0, 0.01, 0.1, 1, and 10 μM. **(E)** c-KIT and phospho-c-KIT levels in M3-transfected cells after imatinib treatment at a concentration of 0, 0.01, 0.1, 1, and 10 μM. **(F)** c-KIT and phospho-c-KIT levels in M4-transfected cells after imatinib treatment at concentrations of 0, 0.01, 0.1, 1, and 10 μM. **(G)** Values of p-cKIT to cKIT are displayed in the line chart for each plasmid at imatinib concentrations of 0, 0.01, 0.1, 1, and 10 μM relative to the individual value at 0 μM.

**FIGURE 5:**
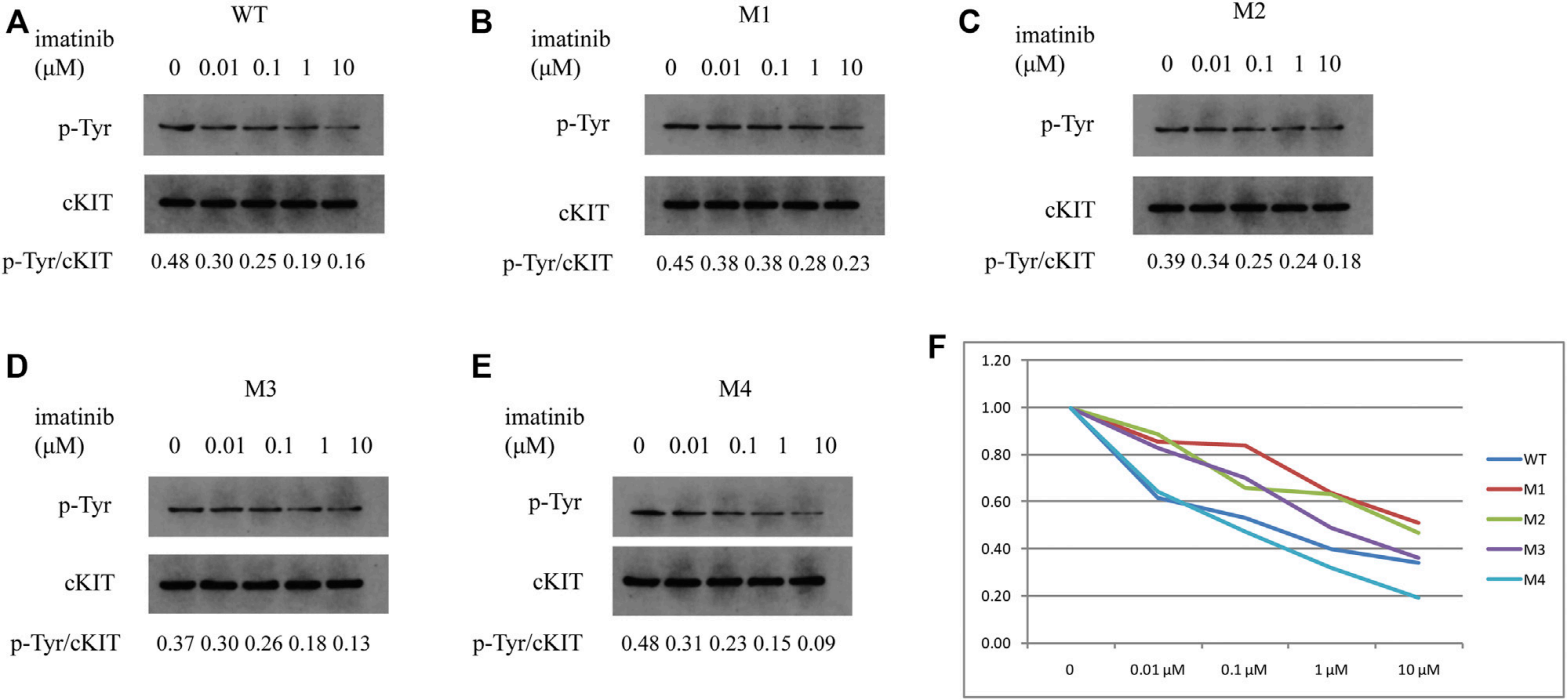
Phosphotyrosine levels were analyzed in SK-MEL-28 cells transfected with various plasmids using Western blotting. p-Tyr and cKIT levels were indicated in cells transfected by wild-type plasmid **(A)**, M1 **(B)**, M2 **(C)**, M3 **(D)**, and M4 **(E)**, respectively, which were treated with imatinib at concentrations of 0, 0.01, 0.1, 1, and 10 μM. The ratios of p-Tyr to cKIT are shown at the bottom of each panel. As shown in the line chart **(F)**, the values of p-Tyr to cKIT were compared to the individual value at 0 μM for each plasmid at the individual concentration of imatinib. p-Tyr denotes phosphotyrosine.

To further demonstrate if the two novel mutations discovered in exon 13 of the *c-KIT* gene could result in imatinib resistance, the monoclonal mouse anti-human c-KIT antibody and rabbit anti-human phospho-c-KIT antibody, as well as anti-phosphotyrosine antibody, were employed to examine c-KIT protein levels following the imatinib mesylate treatment on SK-MEL-28 cells after the transfection of pcDNA 3.1 plasmids, which comprised M1 (c.1668_1679delGTGGAAGGTTGT), M2 (c.1668_1679delGTGGAAGGTTGT/c.1926delA), M3 (c.1936T>G), and M4 (c.1926delA) or wild-type sequence. The relative phosphorylation levels were calculated as the values of p-cKIT to cKIT and p-Tyr to cKIT, as shown in Figures 4, 5 respectively. The results indicated that c-KIT expression was relatively invariable for wild-type or mutant sequences, regardless of whether the cells were activated by SCF, while phospho-c-KIT levels were reduced with the elevated dosages of imatinib mesylate (Figure 4). However, the levels of phospho-c-KIT decreased below the levels of that in cells transfected by wild-type sequences when the dosage of mesylate imatinib reached a certain concentration (0.01, 0.1, 1, and 10 μM) for M3 (c.1936T>G) and M4 (c.1926delA) (Figures 4E–G). For M1 with *c-KIT* c.1668_1679delGTGGAAGGTTGT mutation alone, phospho-c-KIT showed a decreased level dependent on the imatinib mesylate concentration (Figures 4C,G). For M2 (c.1668_1679delGTGGAAGGTTGT/c.1926delA), phospho-c-KIT was shown with the intermediate levels between M1 and M4 at the imatinib concentrations of 1 and 10 μM, followed by the levels no greater than those in cells transfected by the wild-type sequence at 1 and 10 μM (Figures 4D,G). The phosphotyrosine levels decreased with the increased concentration of imatinib, which was close to that in wild-type cells for mutant M3 at 10 μM (Figure 5F). The phosphotyrosine level in mutant M4 was less than that in wild type at the highest imatinib concentration.

**FIGURE 6:**
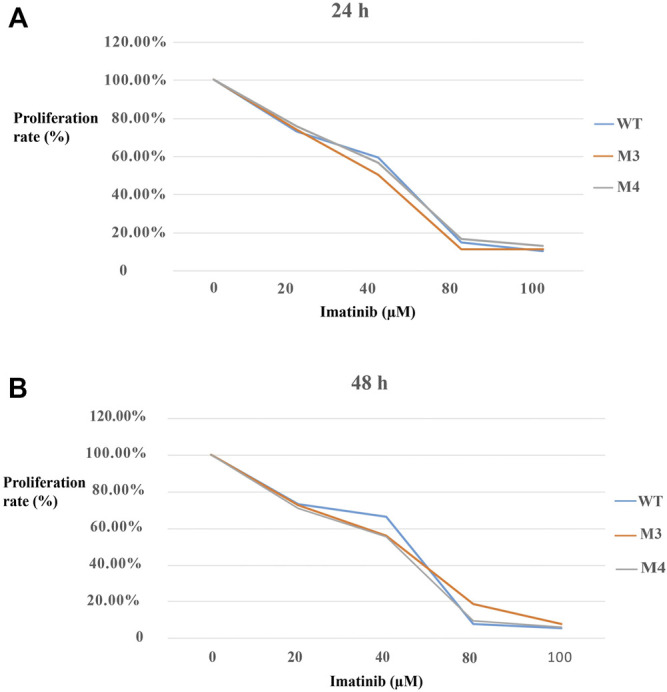
Effects of imatinib at different concentrations (0, 20, 40, 80, and 100 μM, respectively) on the cell proliferation rate of SK-MEL-28 cells transfected with wild-type (WT) or mutated (M3 or M4) c-KIT. **(A)** Proliferation rate decreased with the increase in imatinib concentration when the cells were treated for 24 h. The proliferation rate was equal to the percentage of the mean OD from each treated group divided by the mean OD from MOCK. **(B)** Similar trend was discovered when the cells were treated with imatinib for 48 h.

To investigate the effect of M3 or M4 on cell proliferation, MTT assay was performed to assess the proliferation rate. As shown in Figure 6, the proliferation rate was reduced with the enhanced imatinib concentration regardless of whether the treatment duration was 24 h or 48 h in cells with M3 or M4 mutations. While the imatinib concentration reached 100 μM, the proliferation rate no longer decreased (Figure 6). Furthermore, at this point, the proliferation rate in M3- or M4-mutated c-KIT cells was significantly higher than that in cells with wild-type *c-KIT* (*p* < 0.01) when they were treated with imatinib for 24 h. However, the proliferation rate was still higher, though not markedly (*p* > 0.05), in cells with M3- or M4-mutated *c-KIT* compared to that in wild-type cells when the treatment time was 48 h at the highest imatinib concentration. It is noteworthy that the proliferation rate was remarkably improved in M3-mutated cells treated by imatinib for 48 h at a concentration of 80 µM. The data suggest that M3 or M4 mutation promoted cell proliferation as the imatinib concentration was increased to a relatively high level.

**FIGURE 7:**
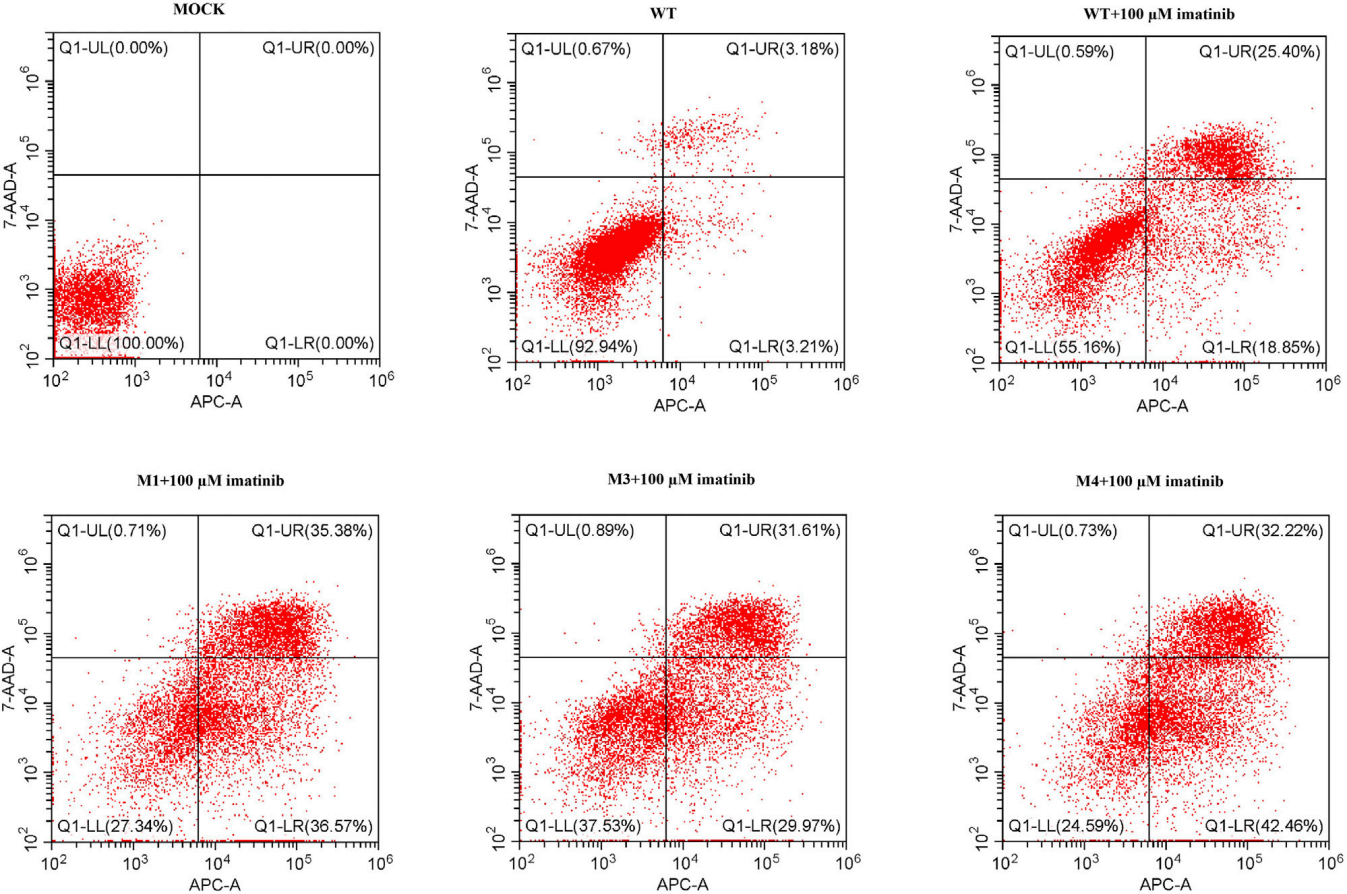
Cell apoptotic rate was assessed by flow cytometry analysis in cells transfected with wild-type or mutated (M1, M3, or M4) *c-KIT* genes after imatinib treatment. Q1-LL indicates the live cells, Q1-LR early apoptotic cells, Q1-UR late apoptotic cells, and Q1-UL dead cells. The data showed that the late apoptotic rate of M3- or M4-mutated cells was lower than that of M1-mutated cells when they were treated with 100 μM imatinib.

To determine the effect of M3 or M4 on cell apoptosis, SK-MEL-28 cells were transfected by plasmids harboring WT, M1, M3, or M4 mutation, and the cell apoptotic rate was measured by flow cytometry. As shown in Figure 7, at the highest imatinib concentration (100 μM), the late apoptotic rate in M3- or M4-mutated cells was lower than that in M1-mutated cells. This indicated that M3 or M4 mutation inhibited cell apoptosis when the imatinib concentration was at a high level.

These results suggest that the novel mutations in exon 13 of *c-KIT* present intolerance to imatinib treatment.

## Discussion

Targeted drugs played a key role in adjuvant or neoadjuvant treatments of GIST patients aiming at the *c-KIT* or *PDGFRA* gene for the high mutation frequency in China. Despite the fact that *c-KIT* mutation occurred at a lower incidence than BRAF and NRAS genes in most of the melanoma patients, it represented 36% of acral lentiginous melanomas in cutaneous melanoma (Ponti et al., 2017). *c-KIT*-targeted therapies enabled the single or combined treatments by receptor tyrosine kinase inhibitors such as imatinib or sunitinib in primary or metastatic melanoma patients. Resistance must be taken into account in the process of using c-KIT-targeted drugs. Mutations in exons 13 and 17 of the *c-KIT* gene account for most of the resistance to imatinib mesylate, which was considered one of the most common receptor tyrosine kinase inhibitors. However, whether the novel mutations in exon 13 of the *c-KIT* gene were responsible for imatinib intolerance in the GIST or melanoma patients remains elusive.

Using Sanger sequencing, patient 1 was found to own a rare mutation c.1668_1679delGTGGAAGGTTGT, causing the amino acid loss of p.557_560del WKVV and gain of p.Q556H mutation in *c-KIT* exon 11, as well as a rare deletion mutation c.1926delA in exon 13, leading to the production of the stop codon (p.K642S*FS). These alterations were present in the biopsy specimen and the surgical specimen of the primary tumor, which might be related to the intrinsic resistance to tyrosine kinase inhibitors. It is thus predicted that intolerance to imatinib mesylate for this patient resulted from the inherent genotype status, instead of that from the acquired drug-induced mutation. It is reported that the latter single-nucleotide deletion causing codon 644 frameshift mutation was only detected in the recurrent duodenal GISTs as the secondary mutation (Vu et al., 2005). Here is the first demonstration of the role of the single-nucleotide deletion in the primary and recurrent tumors of the gastric GIST patient. For patient 2, a point mutation c.1936T>G in *c-KIT* exon 13 produces the missense amino acid alteration p.Y646D, which was not reported in GISTs or melanoma previously. Considering that the effective therapies were lacking and the outcome was routinely poor for metastatic melanoma, determination of the *c-KIT* mutation status was an alternative to the treatment.

In order to further determine whether both rare mutations contributed to the efficacy of receptor tyrosine kinase inhibitors, the wild-type plasmid and the mutant plasmids harboring either c.1926delA or c.1668_1679delGTGGAAGGTTGT or their combination as well as the c.1936T>G point mutation were prepared, as described previously (Isotani et al., 2010). SK-MEL-28, one of the melanoma cell lines, was used as the cell model to be transfected by these plasmids accordingly. The results showed that imatinib mesylate was able to respond to the c-KIT antibody at the phosphorylated state, instead of the unphosphorylated state, for both wild-type and mutated sequences of the *c-KIT* gene. p-cKIT levels were not correlated with SCF stimulation for mutated c-KIT. The c-KIT protein could be activated automatically without SCF for mutated isoforms such as those translated by M3 and M4 mutants. These data were proved by additional literature (Kim et al., 2021). Thus, the levels in those cells would not change upon SCF stimulation. In addition, the cells harboring the mutation c.1668_1679delGTGGAAGGTTGT alone made response to imatinib mesylate in a concentration-dependent way, whereas those with c.1926delA or c.1936T>G produced gradually decreased levels of the phosphorylated c-KIT compared to those in cells transfected by M1, despite the elevated drug concentration. The phosphotyrosine level in M3-transfected cells declined to a level close to that in WT-transfected cells, while the cells with M4 mutation exhibited the c-KIT expression under the level in WT-transfected cells with the increase in imatinib concentration. Our study also showed that M3 or M4 mutation promoted cell proliferation and inhibited cell apoptosis. This is suggestive of the resistance to imatinib mesylate for cells with c.1926delA or c.1936T>G mutation, which is the first demonstration that the mutations are imatinib-intolerant. In addition, according to the published study (Lee et al., 2006), patients with the wild-type c-KIT were able to show certain sensitivity to imatinib treatment; our data were concordant with this. The results showed that the cells with M3 and M4 mutations tended to suppress the phosphorylation level of c-KIT in cells with M1 or M2 mutation, which indirectly mirrored the resistance to imatinib. This was in agreement with the previous report that mutations in c-KIT exon 13 typically represented inhibition by imatinib (Gajiwala et al., 2009). Patients with high phosphorylation levels are prone to descend continuously, while those with low phosphorylation levels have a tendency to fall to the bottom. For that p-cKIT declined significantly at the low concentration of imatinib, it was representative of the low level at the high concentration of imatinib, which on the other hand suggests the very little space to the bottom level. Interestingly, our data also suggest that either c.1926delA alone or concurrent with c.1668_1679delGTGGAAGGTTGT could contribute to imatinib resistance. Therefore, it was reasonable that the tumor of patient 1 had progressed with a shorter interval since imatinib mesylate was given after surgical resection of the primary tumor. At the same time, it implies that the next generation of receptor tyrosine kinase inhibitors such as sunitinib could be the alternative for patients with either of the alterations.

It is worthy to be noted that the metastatic tissue was removed from case 1 after the primary surgery; however, either the *c-KIT* or *PDGFRA* gene was not examined at that time. This could to, some extent, lead to the conclusion that this recurrent tissue was diagnosed as gastric wall plexiform fibromyxoma at the very beginning, which was not concordant with the report that gastric plexiform fibromyxomas were typical of benign tumors free from recurrence or metastasis (Pei et al., 2020; Arslan et al., 2021). This diagnosis would result in different treatments for GISTs. It should be fully realized that sequencing *c-KIT* gene mutations was inevitable in the assisted diagnosis of GIST patients. qPCR acts as a sensitive method to quantitatively analyze point mutations and short sequence deletions, and it was not suitable for examining longer fragment loss. Sanger sequencing or NGS may be an alternative to confirm the presence of *c-KIT* or *PDGFRA* mutations, whereas Sanger sequencing was selected for its cost-effectiveness in this study. Thus, the identification of the novel *c-KIT* alterations in this study provides potential and actionable biomarkers for GIST diagnosis and therapy.

However, limitations were not ignored as well, one of which was that certified GIST cell lines that currently were not available from the cell bank in and out of China. The primary GIST and melanoma cells should be a preferable option. The coding region between exons 11 and 13 represents amino acids from 550 to 663, whereas the phosphorylated c-KIT antibody contains tyrosine 568 and 570. So the plasmids used to express the mutated c-KIT protein could induce the alteration of c-KIT phosphorylation. The mutations in *c-KIT* exons 11 and 13 reside at the cytoplasmic tyrosine kinase domains, which are activated by extracellular ligand dimerization or directly autophosphorylated. The active conformation could phosphorylate c-KIT or other cell growth- or inhibition-associated intermediates, resulting in the sensitivity or desensitivity of imatinib. Thus, it is very important to test this hypothesis in GIST or melanoma-derived cells. This was another drawback of our study. It would be better for the two novel mutations to be introduced into the cells using CRISPR-Cas9 technology to directly target the chromosome, which avoided the background of the wild type. These point out the potential directions for investigation into the c-KIT-inducing drug resistance mechanism.

In summary, our study could provide significant guidance for clinical drug management in GIST or melanoma patients with these mutations in the *c-KIT* gene. However, it is necessary to study a greater number of samples and analyze the frequency of the mutations studied in the patients.

## Data Availability

The original contributions presented in the study are included in the article/Supplementary Material, further inquiries can be directed to the corresponding authors.
